# Natural Products for Drug Discovery in the 21st Century: Innovations for Novel Drug Discovery

**DOI:** 10.3390/ijms19061578

**Published:** 2018-05-25

**Authors:** Nicholas Ekow Thomford, Dimakatso Alice Senthebane, Arielle Rowe, Daniella Munro, Palesa Seele, Alfred Maroyi, Kevin Dzobo

**Affiliations:** 1 Pharmacogenomics and Drug Metabolism Group, Division of Human Genetics, Department of Pathology and Institute of Infectious Disease and Molecular Medicine, Faculty of Health Sciences, University of Cape Town, Anzio Road, Observatory, Cape Town 7925, South Africa; nicholas.thomford@uct.ac.za (N.E.T.); MNRDAN002@myuct.ac.za (D.M.); 2 School of Medical Sciences, University of Cape Coast, PMB, Cape Coast, Ghana; 3 International Centre for Genetic Engineering and Biotechnology (ICGEB), Cape Town Component, Wernher and Beit Building (South), University of Cape Town Medical Campus, Anzio Road, Observatory, Cape Town 7925, South Africa; SNTDIM001@myuct.ac.za (D.A.S.); arielle.rowe@icgeb.org (A.R.); 4 Division of Medical Biochemistry and Institute of Infectious Disease and Molecular Medicine, Faculty of Health Sciences, University of Cape Town, Anzio Road, Observatory, Cape Town 7925, South Africa; 5 Division of Chemical and Systems Biology, Department of Integrative Biomedical Sciences, Faculty of Health Sciences, University of Cape Town, Anzio Road, Observatory, Cape Town 7925, South Africa; SLXPAL001@myuct.ac.za; 6 Department of Botany, University of Fort Hare, Private Bag, Alice X1314, South Africa; amaroyi@ufh.ac.za

**Keywords:** natural products, drug design and development, innovation, automation, computational softwares, bioinformatics, precision medicine, omics, global health

## Abstract

The therapeutic properties of plants have been recognised since time immemorial. Many pathological conditions have been treated using plant-derived medicines. These medicines are used as concoctions or concentrated plant extracts without isolation of active compounds. Modern medicine however, requires the isolation and purification of one or two active compounds. There are however a lot of global health challenges with diseases such as cancer, degenerative diseases, HIV/AIDS and diabetes, of which modern medicine is struggling to provide cures. Many times the isolation of “active compound” has made the compound ineffective. Drug discovery is a multidimensional problem requiring several parameters of both natural and synthetic compounds such as safety, pharmacokinetics and efficacy to be evaluated during drug candidate selection. The advent of latest technologies that enhance drug design hypotheses such as Artificial Intelligence, the use of ‘organ-on chip’ and microfluidics technologies, means that automation has become part of drug discovery. This has resulted in increased speed in drug discovery and evaluation of the safety, pharmacokinetics and efficacy of candidate compounds whilst allowing novel ways of drug design and synthesis based on natural compounds. Recent advances in analytical and computational techniques have opened new avenues to process complex natural products and to use their structures to derive new and innovative drugs. Indeed, we are in the era of computational molecular design, as applied to natural products. Predictive computational softwares have contributed to the discovery of molecular targets of natural products and their derivatives. In future the use of quantum computing, computational softwares and databases in modelling molecular interactions and predicting features and parameters needed for drug development, such as pharmacokinetic and pharmacodynamics, will result in few false positive leads in drug development. This review discusses plant-based natural product drug discovery and how innovative technologies play a role in next-generation drug discovery.

## 1. Introduction

The scourge of communicable and non-communicable diseases and the challenges of finding drug candidates that can treat these diseases with little or no side effects is a huge challenge. Despite the development of drugs for treating and managing diseases such as HIV/AIDS, malaria, hypertension, diabetes and cancer, these diseases continue to plague diverse populations worldwide with significant associated-mortalities. There is need for innovative drug discovery strategies that skew from the current “blockbuster” Pharma R&D strategies. Currently, a viable approach will be to revert to “nature” for answers since it has worked for drug discovery in the past. Anticancer drugs such as Taxol (*Taxus brevifolia*), Vinblastine (*Catharanthus roseus*) and antimalarial drugs such as quinine (*Cinchona* spp.) and Artemisinin (*Artemisia annua*) were all discovered from natural products and are effective in treating these diseases. In the face of global public health challenges, natural products research and development (R&D) potentially plays a pivotal role in innovative drug discovery. 

Plants are found in every habitable environment with most found on land. Faced with many stresses and challenges, coupled with being sedentary, plants have developed many molecules to ward off attacks from animals and environmental insults [1]. These same molecules give plants their ability to give off fragrances, colours and indeed toxicity. Many historical findings report on early use of plants for medicinal purposes [2]. The discovery of medicinal plants by early humans must have been a trial and error exercise necessitated by the need to ease disease manifestations. Before the advent of writing and recording of history, such knowledge was passed through generations through word of mouth. Many plants were recorded in the early years of having medicinal properties and were used to treat many pathological conditions [3,4,5,6,7,8]. Natural products from plants and animals have been the go-to source of drugs especially for anticancer and antimicrobial agents [9,10,11,12,13]. Traditional medicine has been overshadowed by modern medicine as the means of treatment for human diseases [14,15,16]. However, the past few decades have seen an increase in the use of medicinal plants for health promotion and treatment of diseases in many countries including developed countries [17,18,19,20,21,22]. Indeed, many medicinal plant extracts are now used as prescription drugs in numerous developed countries such as the UK, Germany, China and France [23,24].

**Figure 1:**
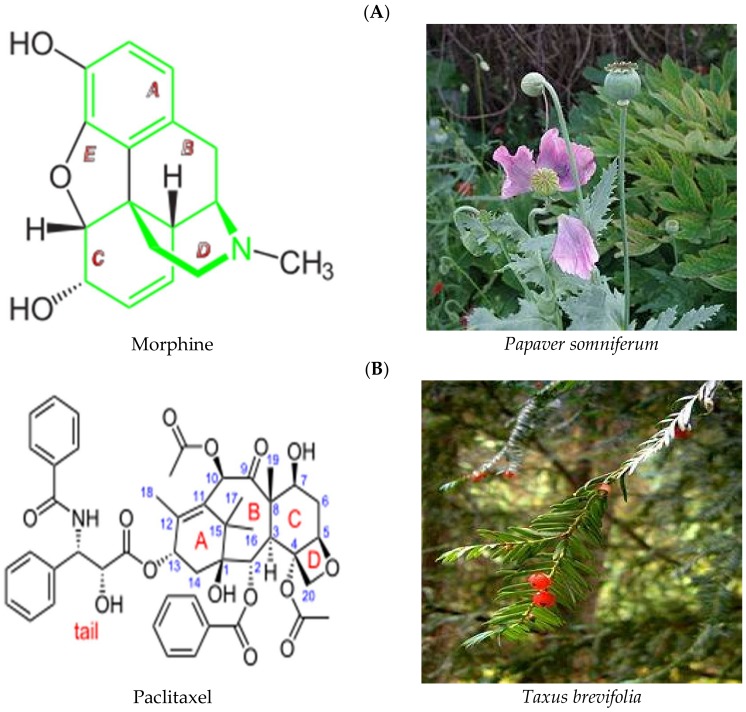
Two examples of successful stories of plant natural products that are being used in hospitals and clinics for disease treatment. (**A**) Morphine is isolated from *Papaver somniferum* also called opium poppy (**B**) Paclitaxel is isolated from *Taxus brevifolia* also called pacific yew. (Images credit: https://en.wikipedia.org/wiki).

About a quarter of all Food and Drug Administration (FDA) and/or the European Medical Agency (EMA) approved drugs are plant based, with well-known drugs such as Paclitaxel and Morphine having been isolated from plants (Figure 1) [25,26]. About a third of FDA-approved drugs over the past 20 years are based on natural products or their derivatives [27,28]. The discovery of penicillin from fungus led to the screening of many microorganisms for potential antibiotics [29]. Indeed, drug discovery from natural products revolutionised medicine. These include tetracycline from *Streptomyces aureofaciens*, artemisinin from *Artemisia afra*, doxorubicin from *Streptomyces peucetius* and cyclosporine from *Tolypocladium inflatum* [27,29,30]. Traditionally, plant extracts are used as concoctions made of combinations of different ingredients. Individually some of the ingredients do not have therapeutic activities, but require their synergistic activities [31]. 

Current challenges to the use of natural products and difficulty in accepting their therapeutic efficacy include: (1) lack of standardization procedures (2) lack of isolation of pure chemical products or compounds (3) lack of elucidation of biological mechanisms and rarely undergoing so-called controlled and (4) documented clinical trials according to “standards”. Historically, there is scientific evidence on the therapeutic efficacy of natural products and as previously mentioned this led to development of some blockbuster conventional medicines. Searching for new drug candidates from natural products is often made difficult by the complexity of the molecular mixtures. The therapeutic activity of plant extracts is usually because of the synergistic and simultaneous action of several chemicals [30,32]. Given the complex nature of many diseases including cancer and degenerative diseases, it is not surprising that the reliance on single compound-based drug discovery has failed to provide effective cures. Plant-based drug discovery therefore must start with a combinatorial approach when evaluating candidate compounds. The advent of novel technologies including quantum computing, profiling techniques, computational biology techniques, big data, microfluidics and artificial intelligence will enable scientists to use a combinatorial approach to harness the therapeutic properties of plant-based natural products and simultaneously study their molecular effects in physiological conditions [33,34]. 

It is however possible that not all components of plant extracts have measurable effects. It has been suggested that one way to improve screening and simplify extracts is through the removal of possible interfering components such as polyphenolic tannins [35]. There are several reported innovative strategies which can be used to achieve this and these include pre-fractionation and extraction methods [36,37]. Indeed, these extraction strategies have resulted in higher hit leads during drug discovery [12,38,39,40]. Innovative extraction technologies including semi-bionic extraction [41], supercritical fluid extraction [42,43,44], microwave-assisted, ultrasonic-assisted and enzyme-assisted extraction [45], molecular distillation methods [46,47] and membrane separation technology [48,49] can be used to extract natural compounds efficiently from plants. These extractions strategies have been shown to have similar simulation to traditional methods allowing the extraction process to get most compounds from the natural product.

Technologies such as high-performance liquid chromatography, nuclear magnetic resonance spectroscopy, mass spectrometry, microfluidics and computational algorithms have seen major advances in the field of medicinal chemistry especially in the 20th century [50,51]. This has allowed the determination of chemical components of plants and their utilisation in drug discovery. High throughput assays using bioreactors and microfluidics systems has led to many drug discoveries using plant natural products. Some of these natural products include opium and morphine [52,53]. Several structural analogues of these compounds are used in clinics and hospitals today. Several new plant-based compounds are emerging as promising anti-cancer remedies. In one of our studies we investigated the anticancer activities of extracts from African lettuce (*Launaea taraxacifolia*), a plant cultivated extensively in Africa, especially West Africa. *L. taraxacifolia* extract caused WHCO1 cell cycle arrest at the G0/G1 phase by affecting differential expression of genes involved in cell cycle regulation, presenting its potential beneficial effects [22]. The medicinal plant *Brucea javanica* (L.) Merr. (Simaroubaceae) has been shown to have many properties and activities. Through both phytochemical and biological investigations, it was shown that *Brucea javanica* (L.) Merr. contains many compounds with medicinal properties. For example the seeds of *Brucea javanica* contain several compounds such as quassinoids that show many biological properties, such as antitumour and antimalarial effects [54]. A well-known malarial drug Artemisinin is a natural product from *Artemisia annua* also known as Sweet Wormwood [55,56]. Artemisinin and its structural derivatives are also used for diseases such as type I diabetes and cancer [57,58,59]. High throughput screening assays face many challenges. For example, the Rio Convention on Biodiversity is aimed at limiting the use of natural products and deals with intellectual property rights. This has the effect of limiting access to natural products as there are fears of extinction of natural species [29,60,61,62,63,64,65].

Current drug discovery strategies and modern medicine discard the use of whole plant extracts and are driven by single compound-based medicine. Taking the whole plant or extracts with no isolation of components as practised in traditional medicine, produces a better therapeutic effect than individual compounds. This is important as most of the plant metabolites likely work in a synergistic fashion or concurrently to give the plant extract its therapeutic effect. Research into the use of whole plant extracts must be done as this will allow scientists to determine the molecular basis of the therapeutic effect of the plant extracts. For example, anti-asthma herbal medicine made from extracts from *Ganoderma lucidum*, *Glycyrrhiza uralensis* and *Sophora flavescens* alleviates bronchoconstriction in an animal model whilst restoring cytokines balance, contributing to longer lasting anti-asthma benefit after treatment [66]. The therapeutic effect only emanates from the synergistic effect of chemical components of the three herbal ingredients [67,68]. The adoption of Good Manufacturing Practises has allowed the increased use of plant-based medicines and many are now undergoing clinical trial for FDA approval [69]. Skroza and colleagues showed that catechin and resveratrol have synergistic effects as confirmed by different antioxidant assays [70]. The same study also showed the synergistic effects of caffeic acid and resveratrol by the ferric reducing ability of plasma (FRAP) antioxidant assay [70]. Another study showed the synergistic effects of ethnomedicinal plants of the *Apocynaceae* family and antibiotics against clinical isolates of *Acinetobacter baumannii* [71]. Several other studies showed the synergistic effect of different plant extracts and conventional drugs including doxorubicin [72]. 

Innovative drug design from natural products is needed to combat global health challenges with the assistance of technological innovation. Most importantly is the need for new and innovative computational and analytical methods to identify chemical components of crude plant extracts in order to identify compounds causing the desired therapeutic effect and optimize extraction to exclude interfering components. Ultimately, more research should be focussing on combinatorial effects of chemicals from plant extracts and not just single compounds. How these combinations affect genes and proteins involved in many cellular processes must be investigated through available “-omics” platforms. Developments in the field of microfluidics and computational analysis have allowed for the designing and testing of plant extract chemicals in drug discovery. Technological advances, such as the development of new analytical and bio-informatic techniques, will aid the design of new structures, the synthesis of these new compounds and the biological testing of such compounds [73,74]. Natural products, offer an endless source of compounds to help in the design of pharmacologically important molecular products [75,76,77]. Below we discuss some of the major innovations currently taking place in these areas. We focus on the need for the use of “-omics” technologies, automation and big data during drug design and testing to allow for the rapid production of drugs and computer aided drug design from plant-based natural products. 

## 2. Multidisciplinary Approach to Natural Products Drug Discovery Using Innovative Technologies

**Figure 2:**
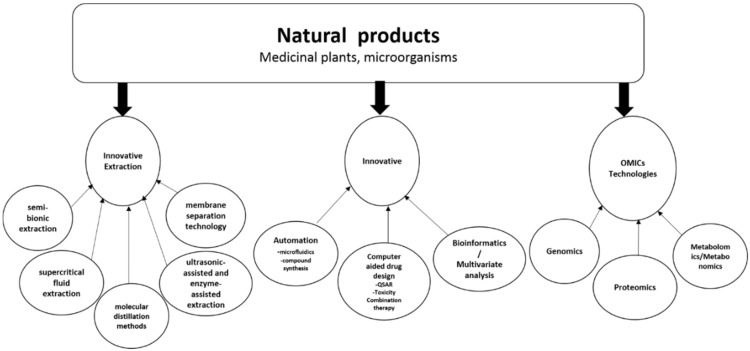
Innovative technologies for natural product drug discovery. Application of these technologies can potentially lead to novel drug candidates from natural products.

Innovative drug discovery from natural products requires a multidisciplinary approach utilising available and innovative technologies to package such natural product compounds for medical practice and drug development (Figure 2). The successful use of such an approach will allow the development of next-generation drugs to combat the ever-increasing health challenges of today and the future.

Most medicinal extract components often work in a synergistic manner to elicit their therapeutic effects so isolating individual components may be counter-productive. Innovative approaches are needed to study and to harness such compounds that can effectively lead to innovative drugs. In addition, a systems biology guided approach provides a different angle in natural products pharma-sciences [78]. This transcends looking for a specific molecule with a specific target and espousing the complete equilibrium of a physiological system undergoing synchronized mechanisms on multiple molecular targets. A systems biology approach coupled with application of available technologies such as genomics, transcriptomics, proteomics, metabolomics/metabonomics, automation and computational strategies will potentially pave the way for innovative drug design leading to better drug candidates. Molecular libraries of lead compounds from natural products R&D will serve as sources of lead compounds/herbal tinctures for innovative drugs. In the application of innovative technologies combined with systems biology, the focus should not be a reductionist approach of trying to source a single active compound but to consider the synergistic effects of compounds. It is important to emphasise that innovative drug discovery from natural products will require a non-reductionist strategy to understand their complex mechanisms of action at the molecular level.

## 3. Natural Products Drug Discovery Research and Development and Omics (Genomics Proteomics and Metabolomics/Metabonomics)

### 3.1. Genomics in Plant-Based Natural Products Identification and Biomarker Identification

The quality, precise identification and reliability in the plant species from which the natural product is obtained and to which the therapeutic properties are ascribed is very critical for successful innovative drug discovery. The use of a different or wrong plant species will likely affect the therapeutic properties due to different compounds and quantities that will be found in the species. Genomic methods are important in establishing an accurate identification method for plants and natural product species [78]. Genomic techniques such as DNA barcoding are established techniques that rely on sequence diversity in short, standard DNA regions (400–800 bp) for species-level identification [79]. DNA barcoding utilising genomics will provide a more robust and precise identification compared to traditional methods of morphological identification and local traditional (vernacular) names [80]. DNA barcoding of natural products has been applied in biodiversity inventories [81] and authentication of herbal products [82,83,84]. DNA barcoding was used in an integrative approach for identification of plant species such as *Amaranthus hybridus* L. and crude drugs recorded in the Japanese pharmacopoeia using *ITS2* or *psbA-trnH* sequence amplification [80,85]. Genomic-based techniques represent an effective platform for natural product identification but different parts of the same plant with similar sequences may have different qualities, clinical utilities and indications due to the diverse conditions under which they grow. 

To show consistency in the species and pharmacological molecules from natural products, bio-farming can be used to ensure consistency after the traditional species have been authenticated through DNA barcoding [86]. Markers developed from species through genomic techniques can be incorporated into DNA chips to provide an effective, high-throughput tool for genotyping and also plant species authentication [78,87]. Gene expression using microarray analysis is an innovative transcriptomic technology that allows a fast and effective analysis of many transcripts [78,88,89]. This transcriptomic analysis makes it possible to concurrently evaluate variations in multiple gene expressions [90]. This represents a robust tool for elucidating the molecular mechanisms of therapeutic natural products and biological networks underlying their pharmacological actions. 

Besides its use in natural products identification, genomics can also be used in natural product or compound targeting. Whole genome sequencing combined with transcriptomic analyses has allowed the exploration of drug or compound targeting as never before. Transcription factor binding sites, protein modifications, alterations of the DNA structure as well as methylation patterns can now be analysed and measured at the genome level [91,92,93,94,95,96]. Several studies including our own have identified deletions, insertions, copy number variations, splicing variants and translocations associated with certain cancers, and in so doing identified new drug targets [97,98,99,100,101,102]. The development of novel and unrivalled technologies, allowing genome-wide analysis, has enabled the unbiased discovery of drug targets. These technologies together with the availability of huge databases of chemicals or compounds have enabled the shortening of the time required for the whole process of drug discovery from drug design all the way to clinical trials [103,104,105,106,107,108,109].

### 3.2. Proteomics in Natural Product Validation and Biomarker Identification

Complimentary to genomic and transcriptomic approaches to quality control and sample variation is the use of proteomic platforms in describing the mechanism of action of many natural products. Proteomic approaches to innovative drug discovery from natural products have the potential to elucidate the protein expression, protein function, metabolic and biosynthetic pathways based on therapeutic effects translating to consistency in quality and profile of the product [110,111]. Approaches such as mass-spectrometry utilising isotope tags and two-dimensional electrophoresis will give insight into quantitative protein profiling which generates quantitative data on a scale and sensitivity comparable to what is generated at the genomic level. Proteomics application has been successfully used in identifying species of Chinese herbal medicine, *Panax ginseng* versus *Panax quinquefolium* [112,113]. The therapeutic effects of natural products can be elucidated using proteomics and imaging techniques to successfully study the metabolism of natural products and their compounds [114,115]. Proteomics is an effective way to elucidate multi-target effects of complex natural product preparations as well as the discovery of multiple compounds and fractions, characterisation of natural products and ultimately a molecular diagnostic platform [78,116].

For natural products to be used as drugs it is crucial that their target proteins be identified. Several methods including affinity chromatography have been in use to identify target proteins with relative success. The advent of technologies allowing for target protein identification without the modification of the natural product has resulted in natural products with increased activity. Such methods include cellular thermal shift assay which is based on the stabilisation of target protein when it binds to its ligand, thermal proteome profiling a method based on the stability of target proteins at high temperatures, bioinformatic-based analysis of connectivity and drug affinity responsive target stability. Due to their many structures and complexity, natural products do show a wide range of biological activities. This is probably due to their abilities to bind to several ligands. Every potential drug will have to be tested for side effects and this is due to its off-target effects. Complex natural compounds with potential target proteins will have to be evaluated properly to identify all its potential target proteins. One of the most utilised methods to identify target proteins and their biological activities is affinity chromatography [117,118,119,120,121,122,123]. This method is a pull-down method in which the natural product is immobilized on a physical solid support [124]. The identification of bound proteins is done using mass spectrometry. Modification of natural products however, can lead to reduced or loss of activity. The development of novel and innovative approaches, devoid of any modification, is paramount for the success of target identification [125,126]. Of late, several methods have been able to identify target proteins using label-free natural products. These new and improved methods measure the responses of natural product-target protein complex to proteomic and thermal treatment [127,128,129]. Using this new approach, it is possible to identify several target proteins for an individual natural product using proteomic analysis [13,130]. 

#### Methods for Target Identification of Label-Free Natural Products

Drug affinity responsive target stability (DARTS) is one of the direct methods used to identify target proteins using label free natural products [124]. This methods takes advantage of the changes in stability of a natural product-bound protein versus an unbound protein when subjected to proteolytic treatment [130]. This method has been used to validate several target proteins for compounds such as resveratrol and rapamycin [129,131]. It is however difficult to use DARTS to identify low abundance protein targets in cell lysates [132]. Another method that takes advantage of ligand-induced changes to target proteins is stability of proteins from rates of oxidation (SPROX) [124,133,134]. This method measures the irreversible oxidation of methionine residues on target proteins [124]. A mixture of candidate drug compound and proteins is incubated with an oxidising agent and guanidinium hydrochloride in order to oxidise methionine. Generated peptides are then analysed through mass spectrometry to evaluate selective methionine oxidation. Analyses of oxidised and non-oxidised methionine-containing peptides versus the guanidinium hydrochloride concentration reveal that proteins bound to ligands show a larger transition midpoint shift than control samples [13,135,136,137]. Indeed, several target proteins of compounds such as resveratrol and cyclophilin A were verified using SPROX [133,137,138]. This method however requires highly concentrated proteins for analysis. Modifications of the SPROX method, named stable isotope labelling with amino acids in cell culture (SILAC)-based SPROX is an improvement of the original method and has the advantage of covering more target proteins [130,139,140,141,142,143,144]. This method is limited to only identifying of methionine containing proteins. 

Cellular Thermal Shift Assay (CETSA) is a recently introduced method based on stabilisation of a target protein by binding to its ligand [145,146,147]. Cell lysates and intact cells are treated with the candidate drug compound and heated to several temperatures and target protein is separated from destabilised protein and analysed by Western blot analysis. Shifts or changes in melting curves are detected when ligand–target interactions are plotted against temperature. This method has been useful in identifying target proteins of many anti-cancer therapeutic agents such as raltitrexed and methotrexate [145]. The advantage of this method is the obvious use of intact cells with no need for treatments or preparations. Due to the use of Western blot step it can be very selective. Some target proteins with unfolded binding sites, however, may not be detected. In addition, due to non-specificity of some antibodies used in Western blot step, off-target proteins may also be identified as false positives. Thermal Proteome Profiling (TPP) is an advanced modification of the CETSA method. This method identifies target proteins displaying thermal stability at high temperatures induced by ligand binding and the use of mass spectrometry to measure ligand–target protein interaction at cellular level [148,149,150]. This method uses isobaric mass tagging in for high resolution mass spectrometry. Most expressed soluble proteins will show melting curves resulting in the identification of both target and off-target proteins [13,127,148,151,152]. By identifying off-targets TPP can be used to study possible side effects of candidate drug compounds [150]. This method is very costly and is labour intensive.

Small interfering RNA and short hairpin RNA are obvious choices for target gene manipulation to functionally validate target protein and natural product interactions [153,154]. By knocking down target protein using interfering RNA it is possible to study off-target effects of candidate compounds. Recently clustered regularly interspaced short palindromic repeats-Cas9 (CRISPR-Cas9) genome editing approaches have been used to overcome off-target effects of candidate compounds and to delineate how many natural compounds work [155,156]. CRISPR-Cas9 based genome editing combined with high throughput sequencing and computer-based mutation analysis, referred to as DrugTargetSeqR, has been used to study drug resistance and for validation of several anti-cancer therapeutic agents [157,158,159].

### 3.3. Metabolomics and Metabonomics Approach to Natural Products Drug Discovery

**Figure 3:**
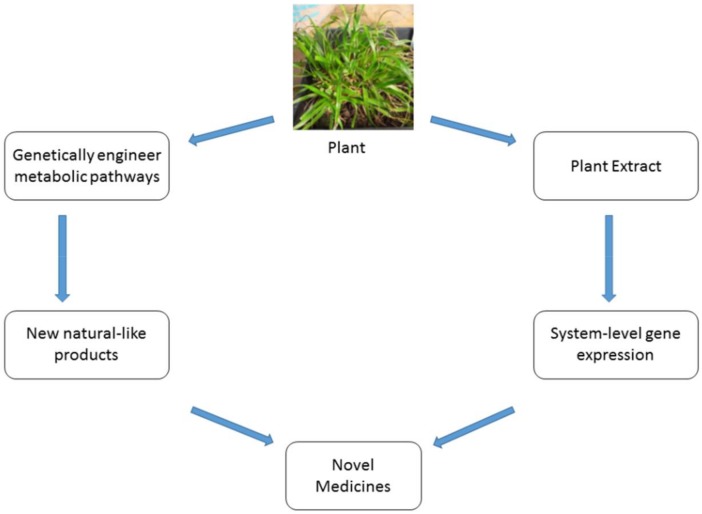
Exploiting the properties of plant extracts in the development of novel medicines inspired by compounds found in medicinal plant extracts.

Untargeted metabolomics and metabonomics approaches of discovering compounds of therapeutic interest from natural products have the potential to lead to innovative drugs for global health. Metabolomic profiling of natural products seeks to identify and quantify the complete set of its characteristic metabolites [160,161] while metabonomics broadly aims to evaluate the global and dynamic metabolic response of living systems to biological stimuli or genetic manipulation [162,163,164,165]. Drug discovery has traditionally focussed on metabolomics to identify metabolites but recently, the term metabonomics (although used interchangeably) has been reviewed to incorporate a systems biology guided approach to study the functions and perturbations of a biological system following a pharmacological effect. This elucidates a complete biological mechanism of both the natural product and its effect on a living system (Figure 3). 

Metabolomic profiling of natural products using technologies such as ultra-performance high performance liquid chromatography–quadruple TOF MS (UPLC–MS) has enabled identification of compounds that confer therapeutic properties on herbs such as *Newbouldia laevis*, *Cassia abbreviata*, *Hyptis suaveolens* and *Panax* herbs [166,167,168]. As a quality control measure and to show consistency in species usage, metabolomics has been used in identification of processed *Panax* species (*Panax ginseng* and *Panax quinquefolius*) using Nuclear Magnetic Resonance (NMR) based metabolomics, UPLC–QTOF MS and multivariate statistical analysis [169]. Metabonomics approach to profiling natural products for drug discovery has been hailed as a critical phenotyping tool. The systems biology approach of this technique positions the profiling of natural products in an all-inclusive manner in terms of metabolite and biology systems effect (Figure 3). Metabolomic and metabonomics profiling using NMR, MS and UPLC can potentially elucidate the pharmacodynamic, pharmacokinetic and toxicological value of natural products. 

### 3.4. Big Data in Drug Development for Natural Product Drug Development and Precision Medicine

Omics analysis, like genomics, transcriptomics, proteomics, metabolomics and metabolomics, results in a generation of a complex multivariate dataset that requires computational and chemometric tools for interpretation. The use of computational platforms such as bioinformatics and multivariate statistical tools, will allow the application of omics multidata to elucidate pathophysiological effects, target specificity and molecular effects, as well as elucidate the pharmacodynamic, pharmacokinetic and toxicological characterisation of natural products and their compounds. Applications used during the drug discovery process such as docking and virtual screening can make use of novel machine learning algorithms such as deep learning. Machine learning methods can be used for virtual screening of thousands of compounds allowing the utilisation of data from high throughput screening [170,171]. 

Computer-based screening of candidate compounds for drug discovery makes use of big databases especially to identify compounds of similar activity. Similarity in structure is equated to similarity in biological activity, with results not always supporting this idea. Knowledge of the chemical structure of candidate compounds together with knowledge about the target protein is utilised to study possible interactions between the two. Transcriptomic data, used as gene signature, can be used to compare differences and similarities in response to candidate compounds [172,173,174,175,176]. For example, the connectivity map allows scientists to associate disease-associated gene signatures with drug signatures resulting in the identification of drugs that can potentially reverse the disease gene signature [177,178,179,180,181]. Generating a lot of data can have the consequence of losing the ability to understand its meaning. Big data must be useful and put into action. For big data to be useful during drug discovery it must be summarised into a little actionable information [182,183,184]. There are several data sources used for drug identification. These include ChemBank, PubChem, ChEMBL, DrugBank, UniProt, STITCH and the NIH Small Molecule Repository [185,186,187,188].

Connectivity Map (CMap) is a bioinformatic application that allows the study of diseases at the molecular level with the help of computers [189,190,191]. Established by the Broad Institute, the CMap is a collection of transcriptional data of many compounds-treated human cells [172,173,174,175,192]. The CMap associates gene expression signatures with compounds, genes and disease response allowing for its utilisation to show connections between compounds with the same modes of action and same physiological processes [172,173,174,192]. The CMap also allows associations to be made between diseases and drugs. The same pattern-matching analysis can be used for natural products, gene expression and diseases [173,174,175,176]. 

Using electronic databases of chemicals and protein targets and clinical data such as patient to patient variations in response to treatment, several strategies are being employed to reduce the cost of drug development and to increase the speed at which drugs are developed [193]. There are obvious challenges to the efficient development of drugs and these include the lack of models that can recapitulate the human body properly in terms of response to candidate compounds, the heterogeneity of individuals in terms of their response to candidate compounds and the inability to analyse biological processes properly during testing of candidate compounds [194,195,196]. Despite a heavy investment in research and development, most candidate compounds show weakened efficacy as the stages of drug development move towards clinical trials [197,198,199,200,201]. This strategy whereby information is collected without applying a hypothesis or any bias, analysed and then used to come up with new and innovative ideas is called Big Data [202,203,204,205,206]. Big data is now integrated with compound chemical structures, protein structures, compound toxicity and clinical trials and this has led to the development of complex algorithms needed for such analysis [207,208]. 

One major challenge with the use of big data-driven drug discovery is the relative low presence of similar somatic alterations in cancer patients enrolled in a study. This is caused by tumour heterogeneity, a chief cause of chemoresistance and drug treatment failure. A challenge to scientists using big data to inform drug development and testing is how to integrate a lot of information into a meaningful and manageable unit. For “omics” data to be meaningful and to revolutionise clinical medicine, clinical phenotype data has to be integrated with genomic, transcriptomic, proteomic and epigenomic data [33,193,209]. 

## 4. Automating Natural Product Drug Discovery

Automation is usually associated with negative feelings, with many people associating automation with loss of jobs and unfounded consequences such as robots taking over the world. In terms of drugs discovery automation, however, it has been used successfully to speed-up the process. Indeed many pharmaceutical companies already have high-throughput assays robustly used in the drug discovery process [210]. The design of most synthetic compounds is aided by computers using various softwares, as well as the synthesis of the compounds. Examples of softwares used during drug design include ADAM and EVE, used in target and hit finding [211,212]. New softwares and devices are being made to reduce problematic false positives an also to reduce material consumption during compound design, synthesis and biological testing [213]. For example, integrated microfluidics systems, with the ability to handle liquids and heat necessary for during-synthesis analyses and purification, are being designed by laboratories and pharmaceutical companies, for compound screening and synthesis of compounds [214,215]. This has allowed testing of several hypotheses within days. Even more advanced technologies through the use of artificial intelligence (AI) and ‘organ-on-chip’ technologies are now fully integrated in the drug discovery process, aiding scientists during drug design and optimisation of the drug discovery process [213,216,217,218,219]. All these technologies have allowed the reduction of human mistakes and bias commonly made during drug design and optimisation, reduction in the amount of candidate compound needed for the testing, have reduced the time needed for testing of candidate compounds to days and allowed the recapitulation of disease biology more effectively than in vitro assays [220,221]. Many times, innovation and technological advances have raised false hopes and never lived up to expectations. Automation and innovation in drug discovery must be fast, but also sustainable in the long run [33,222]. 

Several factors are taken into consideration during compound or molecule design. These include absorption, distribution, metabolism, excretion and toxicity (ADMET) properties and the final biological activity of the products. Thus, the optimisation of the drug discovery process is multidimensional. In the end, a balance has to be achieved in order to get the best in terms of compound activity and properties [213]. Automation will allow scientists to make the best decision regarding the best compound design with relevant biological activity whilst at the same time having desirable ADMET properties. Several concepts such as the diversity-oriented synthesis (DOS) and biology-oriented synthesis (BIOS) have been developed over the past few years to aid compound design and increasing compound collections with new chemical structures and constituents [223,224,225,226,227,228]. An even advanced concept is the function-oriented synthesis which seeks to mimic the function of a promising compound in order to get simple scaffolds and make their synthesis easier and simple [213,229,230]. Several automated compound generators that use deep learning techniques have been made and have allowed automated analysis of generated compounds to obtain even better designs of compounds with desired properties and biological activity [216,217,231,232]. Although deep learning models are used mainly to predict drug-target interactions and in the generation of new molecules, these models are also useful to predict ADMET properties of novel candidate drugs [233,234]. Several deep learning models have also been used to predict the binding affinity for candidate compounds during drug discovery [234]. Several compounds based on the imidazopyridine scaffold have been synthesised using automated computer-assisted de novo design resulting in the discovery of many ligands for G protein-coupled receptors antagonists [213,235,236]. It is also possible to use a virtual library enumeration parallel to target panel prediction to design a compound library and building block selection. Using integration of computational activity prediction and microfluidics-assisted synthesis enabled scientists to identify ligands with different binding profiles [236,237,238,239]. Thus, microfluidics synthesis and computer-aided target prediction can be used to generate bioactivity-focussed compound libraries rapidly and efficiently [235]. 

An important part of automation of compound synthesis is the availability and use of building blocks and chemical reactions that can result in diverse by-products. The use of small volumes of the starting compounds and compact synthesis coupled with in-line purification and analyses ultimately led to the development of novel machines to synthesise complex structures recapitulating the biosynthesis of most natural compounds [240,241,242]. Importantly, 3D printing can allow for the building of different microfluidic devices with several sophisticated and specialised algorithms to monitor product synthesis. 3D printing is very important for microfluidics platforms as most microfluidics systems are custom made for a specific function. Some of the latest approaches using automated robotic synthesis are remotely controlled making it even more efficient [243,244]. Some automated compound synthesis approaches are very versatile with only a small set of building blocks being needed to generate a diverse group of by-products [245]. Microfluidics based synthesis of compounds allows the continuous synthesis of compounds and not batch-wise. Cytochrome P450-catalysed drug oxidation can now be simulated meaning that in future on-chip chemotransformations of compounds can replace in vitro metabolite identification [246,247]. Microfluidics synthesis of compounds, coupled with in-process analysis and purification, is revolutionising drug discovery automation [248]. Besides the obvious avoidance of human exposure to chemicals and dangerous solutions, microfluidics also allows the use of minimum amounts of compounds and reagents [249,250,251]. Given that most animal models are very poor predictors of human response to drugs and biological testing, microfluidic systems can aid in recapitulating human- and species-specific functions by incorporating organoid-based approaches. This allows for the generation of physiological relevant environments within the microfluidic devices and these can be stable over some time [252,253]. Several systems incorporating cancer cells or 3D cancer models have been developed and allow for the recapitulation of human tumours and their microenvironments [254,255,256]. Several constraints do exist for continuous flow systems such as microfluidics synthesis of compounds. The synthesis and eventual deposition of reactive reagents and by-products brings about the danger of fluidic surfaces instability as some of the reagents and solutions used in compound synthesis are incompatible with the microfluidic systems. In addition, clogging of channels of the microfluidic system is a major problem. 

Several integrated microfluidics-assisted synthesise and test platforms are now available combining the reagent and compound selection and can adapt based on materials available for the subsequent steps during synthesis and testing of compounds. Several computational tools and networks (containing millions of reactions and pathways for compound synthesis) used for automated compound synthesis have been developed and aid in finding the optimal and innovative route to compound synthesis [257,258,259,260]. Drug design with the help of artificial intelligence is a requirement to have a sustainable drug discovery process [261,262,263,264]. Hypothesis generation if done by machines can result in the designing of compounds using several criteria at the same time. Such criteria can be biological activity, side-effects and synthesizability. Machine-guided hypothesis or generation of compound structures is also much faster and can generate different designs at the same time. Artificial intelligence is therefore an enabling technology, aiding the scientist in pattern recognition and can be optimised to do pattern recognition [265,266,267]. 

## 5. Computer-Aided Drug Design from Natural Products

Synthetic compounds with structures inspired by natural products can help solve many global health challenges while in many instances some of the new synthetic compounds would have been discarded as not suitable for drug designs. The so called “rule of three” and “rule of five” criteria often used for decision making with regard to drug leads is too strict and some of the new designs would have been failed [268,269,270,271,272]. In fact, many of the guidelines used during drug designing show human bias and therefore are limited in their scope and effectiveness, especially when applied to natural products [269,270,271,272]. Many therapeutic synthetic compounds have been developed using computer-aided designs and these include several anticancer agents [273,274,275,276]. The Scaffold Hunter software for example was used to simplify complex natural products to generate virtual fragments of small chemically attractive molecules [277]. The simple molecules visualised by such computational software must retain the same biological activity as the mother compound. Indeed, this method was already used to identify inhibitors and activators of pyruvate kinase [278]. However, it is also possible that natural-product derived simple molecules will exhibit weaker activities than the parent compound [275,276]. The PASS software has been used to predict the biological activities of simple structures or chemical structures obtained from the mother compound with considerable success [279,280]. The PASS software has predicted the anti-tumour activities of several marine alkaloids [278,279,280]. 

Indeed several individual compounds from St John’s wort were also predicted rightly to have cytochrome P450 modulating effects [278]. Several computational softwares, databases and web servers have been developed that can predict compound-target associations. Most if not all of these softwares use the similarity of new compound to known drugs to infer target and normal ligand–receptor docking. In the absence of any similarity between new compound and any known drug, the SPIDER software can compare computed features between natural products and new compound to predict the target of the new compound [281,282]. The identification of G-protein coupled receptor ligands was one of the success stories of the SPIDER software [282]. The use of computational drug design and target prediction is now tangible and will continue to influence drug development in the near future. However only previously studied targets or proteins can be predicted. Computer based quantitative structure activity approaches can be employed in natural product drug discovery to explain the molecular basis of their therapeutic values and to predict possible derivatives that would improve activity [283]. The positive aspect of computer-based drug designs is that it guides optimization of lead compounds as to whether to increase their affinity or pharmacodynamic and pharmacokinetic properties. 

New systems are being developed in order to detect candidate or lead compound toxicity at early stages of drug discovery [284,285,286,287]. Strategies employing in silico methods can be used to detect drug toxicity early on along the drug discovery process. Such approaches if combined with in vitro and in vivo biological testing can drastically decrease the time and cost of drug discovery and improve safety evaluation. Quantitative structure–activity relationship models aim to understand the relationship between the structure of a compound and its toxicity [288,289,290,291]. To understand the possible accumulation of the drug and its metabolism properties such as adsorption, distribution, metabolism and excretion must be evaluated [292,293,294,295]. To compound the issue of candidate compound toxicity, one has to consider environmental toxicity. So, during drug discovery the potential risk of having a drug in the environment must be addressed. These candidate compounds or lead compounds may have toxic effects on other animals. 

The advent of advanced technologies has allowed scientists to discover the magnitude of tumour heterogeneity and the different patients’ responses to treatment [296,297,298,299]. Drug discovery however is based on the “one drug-one target” strategy. It is a fact that combination therapy is the gold standard nowadays. Thus, drug design must take a combinatorial approach, where two or more drugs either target the same pathway or act synergistically to achieve a cure. Conventional chemotherapeutic agents may be combined with targeted therapies such as kinase inhibitors [300,301,302,303,304]. Now, a few approaches for computer-based screening for combinatorial drug design and treatment are under development. 

## 6. Natural Products and Precision Medicine

**Figure 4:**
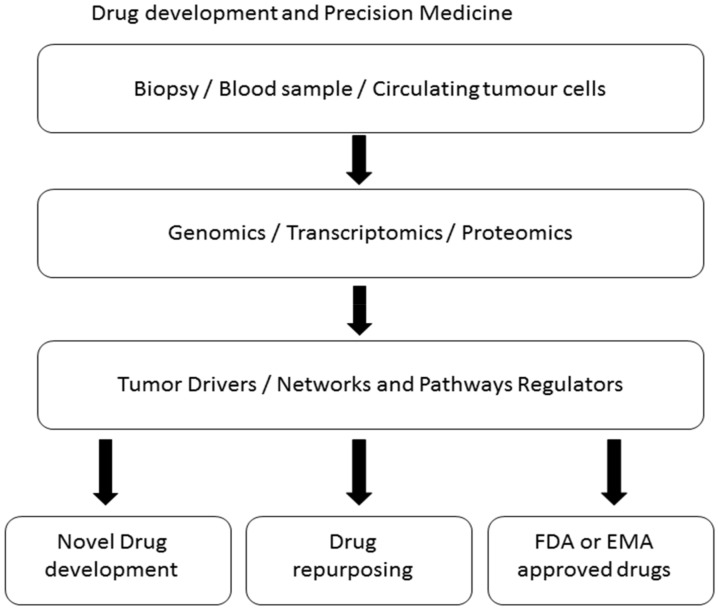
Precision therapies in oncology can be designed to only affect cancer cells. Biomarkers can be identified through next generation sequencing, gene expression profiling and proteomics. Drivers and regulators of important pathways involved in cancer cell proliferation, survival and chemoresistance can be identified. Only with this knowledge can the development of novel drugs be achieved.

The past few years have seen genomics informing drug discovery but overall the clinical efficacy of the resultant drugs has been poor. This is largely due to the complex nature of diseases. Advances in technological and analytical tools used in genomics now allow for the rapid identification and interpretation of genetic differences driving patient specific features of disease (Figure 4) [33,222]. Precision medicine would then target these specific features to obtain a cure. At the heart of the Human Genome project lies the need to understand how genetics impacts disease and vice versa. For oncologists and cancer scientists, how genetics can transform drug discovery has generated a lot of excitement. The rapid development of many new techniques now allows the analysis of patients’ and healthy individuals genomes (Figure 4). Importantly it is possible to link a patient’s genome and clinical presentation [305]. Investigations of whether specific proteins are drug targets culminated in many drugs in use today. Over time however, productivity in terms of drugs produced declined as there were no more definite new drug targets. The “gene to screen” approach was based on the realisation that genes expressed within a cell are the main contributor to the overall cellular phenotype [306,307]. Genome-wide association studies (GWAS) is a cost-effective and unbiased way of genotyping and comparison of genomic variations between patients with disease and healthy individuals. GWAS has led to the identification of genetic determinants of diseases and underlying mechanisms driving disease development.

## 7. Conclusions

The low success rate of drug discovery requires a paradigm shift for innovative drug development strategies. Innovative drug discovery starts by deriving inspiration from natural products for effective treatment of disease conditions. The relevance of natural products in providing innovative drugs to find solutions to communicable and non-communicable diseases cannot be over-emphasized. Technological advances have made it possible to understand the profiles of these complex natural products with the potential to discover new drugs for use. An impressive number of blockbuster drugs have been isolated or synthesized from natural product lead compounds. This positions natural product drug discovery as a very successful strategy for the development of novel therapeutic drugs. In this era of advancing scientific technology, innovative drug discovery from natural products will potentially increase the success rate of new therapeutic moieties. Natural product drug discovery stands as a major contributor to solving global health challenges and achieving sustainable development goals on health.
